# Occlusal Plane Modification in Clear Aligners Treatment: Three Dimensional Retrospective Longitudinal Study

**DOI:** 10.3390/dj11010008

**Published:** 2022-12-27

**Authors:** Domenico Ciavarella, Carlotta Fanelli, Carmela Suriano, Angela Pia Cazzolla, Alessandra Campobasso, Laura Guida, Michele Laurenziello, Gaetano Illuzzi, Michele Tepedino

**Affiliations:** 1 Department of Clinical and Experimental Medicine, Dental School of Foggia, University of Foggia, Via Rovelli 50, 71122 Foggia, Italy; 2 Department of Biotechnological and Applied Clinical Sciences, Dental School of L’ Aquila, University of L’Aquila, Edificio Delta 6, Via Lorenzo Natali, 67100 Coppito, Italy

**Keywords:** clear aligner appliance, orthodontic appliance, occlusal plane, vertical dimensions, bite force

## Abstract

The purpose of the present study was to evaluate: (i) maxillary occlusal plane changes after clear aligners therapy with a 3D measurement technique; and (ii) as a secondary outcome, if such changes were correlated to the patient’s 1axilla-mandibular divergence. 3D maxillary models of 32 patients (7 males and 25 females; mean age 22.3 +/− 3.4 year) treated with clear aligners were analyzed. The angle (α) between a reference palatine plane and a maxillary occlusal plane was measured. Five angular cephalometric measurements (NSL/MP; PP-OP; OP-MP; PP-MP; PFH/AFH%) were performed and related to Δα. The subjects were further divided into three groups according to facial divergence. After aligner treatment, Δα increased in hyperdivergent patients and decreased in hypodivergent patients (*p* < 0.05). Δα showed a significant positive correlation with NSL/MP (rho = 0.44) and negative correlation with PFH/AFH% (rho = −0.53). Aligners treatment produced a counterlockwise rotation of the maxillary occlusal plane, even if this rotation occurs differently depending on divergence.

## 1. Introduction

Clear Aligners Treatment (CAT) is widely used, especially in adult patients, due to advantages, such as better periodontal health [1], a lower risk of apical root resorption [2] and white spot lesions [3], comfort and aesthetics [4], a lower pain level [5], and a positive impact on the patient’s quality of life [6,7]. Progress in material properties and biomechanical protocols lead also to an acceptable clinical effectiveness in orthodontic movements, such as controlling anterior intrusion, posterior bucco-lingual inclination and molar distalization of approximately 2.5 mm [8].

On the other hand, the limits of CAT are related to patients’ compliance and less effectiveness in controlling complex tooth movements, such as anterior extrusion, severe rotations (canines and premolars), molar up-righting, arch expansion and extraction space closure, that can be effectively achieved with conventional fixed buccal [9] or lingual appliances [10].

A particular note needs to be mentioned regarding intrusion movement during CAT: it is known indeed that posterior teeth are easily intruded with clear aligners, an effect that is usually explained with the so called “bite block effect”. The thickness of the aligner plastic combined with occlusal forces leads to the intrusion of the posterior teeth over the course of treatment. Evidence of the bite block effect is provided by the occurrence of posterior open bites in patients after CAT [11,12]. It can be questioned if this effect could systematically determine a change of the occlusal plane (OP) in these patients. It is known, for example, that the treatment with multibracket fixed appliances tends to extrude teeth, increasing the mandibular plane angle. Moreover, it is possible to modify the OP deliberately with adequate techniques and that auxiliaries, such as inter-maxillary elastics for the correction of a dental sagittal discrepancy, determine a variation of the OP [13,14]. 

Despite the constantly growing usage of clear aligners, there is a lack of studies investigating the effects of these appliances on occlusal plane changes and on the masticatory muscles, which role seems to be important in such intrusion of the posterior teeth. Only few studies investigated the muscular response to the prolonged wear of clear aligners [14], mostly focusing on the reduction of myofascial pain or bruxism [15,16]. However, due to the importance of the control of the OP during orthodontic treatment, it would be of great clinical interest to determine analytically the changes on the OP that can be produced by CAT. Furthermore, if the theory of the “bite block effect” is accepted, the force amount and the activation pattern of the masticatory muscles could be an important variable; therefore, different effects in patients with different facial divergence could be expected, since different bite forces are observed in these facial types.

The aim of the present research was thus to evaluate the changes of the OP in adult patients treated with clear aligners, through a bi-dimensional and a three-dimensional assessment. Moreover, a secondary outcome was to evaluate the effect of a hyperdivergent, a normodivergent, and a hypodivergent skeletal pattern on the observed changes of the OP.

## 2. Materials and Methods

A total of 32 Caucasian patients (7 males and 25 females; mean age 22.3 SD ± 3.4 years) were retrospectively enrolled in this study. The present protocol was designed following the recommendations of the Declaration of Helsinki from 1975 and subsequent revisions. All the participants gave their written informed consent to participate. A power analysis (G*Power 3.1.9.2, Franz Faul, Universitat Kiel, Germany) revealed that to detect a large effect size of 0.5 with a correlation test, considering an alpha of 0.05 and a power of 0.93, 31 subjects would be needed [17]. 

The participants were recruited adopting the following inclusion criteria: non-growing patients at the beginning of treatment (selected using the cervical vertebral maturation method, C6 stage) [18], non-extraction treatment, treatment with a clear aligner system (Dooris^®^) with a thickness of 0.75 mm, treatment involving a sequence of 15 aligners or more. The exclusion criteria were: use of auxiliaries in combination with clear aligners, periodontal disease, temporo-mandibular disorders, maxillo-facial congenital syndromes, no implants or root canal therapies before and during orthodontic treatment. 

All the selected patients were instructed to wear the aligners at least 22 h per day and were treated with the same aligner system and the same protocol, involving monthly visits, the change of aligners every 15 days, and a mean treatment time of 13 ± 5 months. 

The patients enrolled in the present study presented a skeletal class I or II with a moderate misalignment in the anterior sextant of the dentition. The treatment setup invoved no movements on the vertical plane (i.e., extrusion or intrusion of molars or incisors). 

### 2.1. Digital Analysis

**Figure 1:**
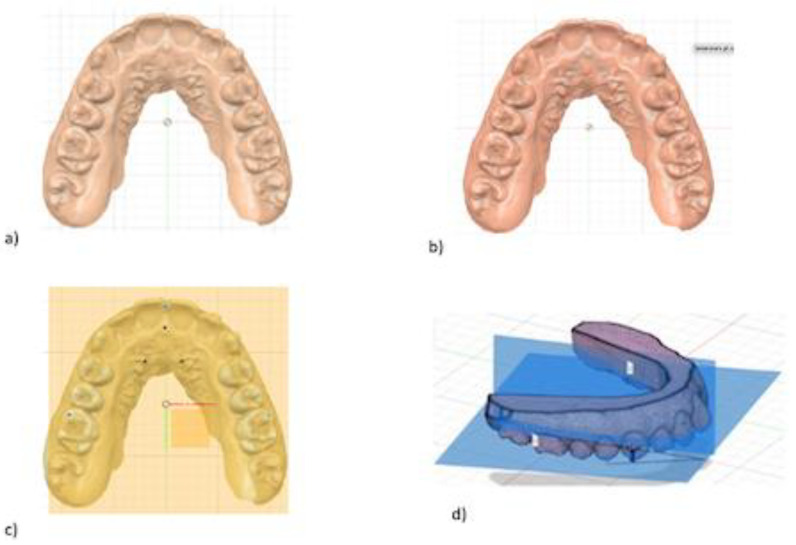
Digital procedure to evaluate the variation of occlusal plane. (**a**) occlusal view of maxillary stl model; (**b**) three points insertion of reference palatine plane; (**c**) maxillary occlusal plane passing through central incisors contact point to mesio-buccal cusps tips of maxillary first molars; (**d**) sagittal intersection between the two planes generating angle α.

A digital impression of the maxillary and mandibular arches was taken before and after treatment, using an intraoral scanner (TRIOS; 3Shape, Copenhagen, Denmark), following the manufacturer’s instructions. The standard triangle language (STL) files were imported into a dental CAD software (Fusion 360^®^; Autodesk Ink) and virtual models were generated. Three hard- and three soft-tissue landmarks were highlighted on the maxilla, and two reference planes were traced: the Maxillary Occlusal Plane and the Reference Palatine Plane, respectively. Each plane was defined by three points as follows: the first plane passing through the bilateral first palatal rugae and the incisive papilla; the second passing through central incisors contact point to mesio-buccal cusps tips of maxillary first molars (Figure 1).

The intersection between the two planes generates the alpha angle α (Figure 1) measured by the same operator using Fusion 360 software. This angle value was measured before and after CAT in order to obtain two values: α0 (pre-treatment scan) and α1 (post-treatment scan). The variable Δα (α1-α0) was assessed. 

### 2.2. Cephalometric Analysis 

Lateral cephalograms were taken (CS 81003D; Carestream Health, Rochester, NY) in habitual occlusion, with relaxed lips and in natural head position and then imported into FastCeph software (Caes software, Grottaferrata, Italy). Subsequently, cephalometric records were digitally traced in the FastCeph software (Caes software, Grottaferrata, Italy). To estimate the error of the method, cephalometric radiographs were selected randomly and reanalyzed 30 days later by the same examiners (C.F. and C.S.). The following planes were traced on lateral cephalograms: (a) Sella-Nasion plane (NSL), the line joining Nasion and the center of Sella turcica; (b) Palatal plane (PP), the line drawn from anterior nasal spine to posterior nasal spine; (c) Mandibular plane (MP), the line passing from Gonion to Menton; (d) Occlusal plane (OP), the line passing from the incisal edge of U1 to the midpoint of the U6 on the occlusal surface. The following five parameters were selected and measured: a) NSL/MP; b) PP-MP; c) PP-OP d) OP-MP (e) PFH/AFH% (Table 1).

### 2.3. Statistical Analysis 

**Figure 2:**
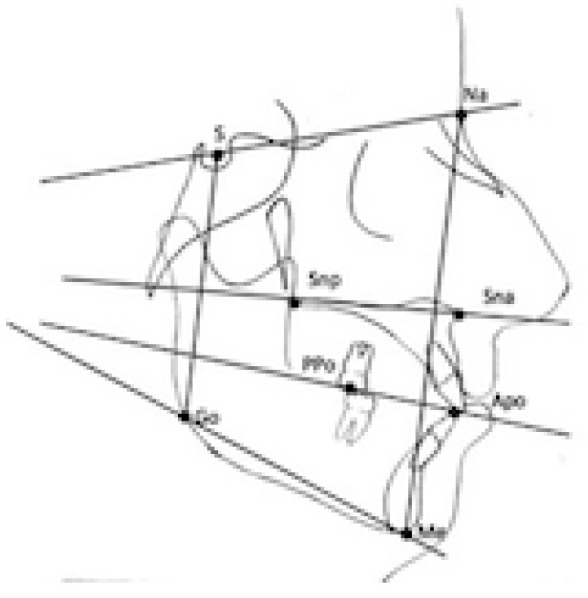
Cephalometric parameters considered.

The collected data were analyzed using the standard statistical software package (GraphPad 6 software.). Descriptive statistics of mean, standard deviation (SD) and standard error of means (SEM) were calculated. The Gaussian distribution of data was evaluated with a Shapiro–Wilk normality test. A Pearson’s correlation test was carried out to evaluate the presence of a statistically significant correlation between Δα and the cephalometric values describing facial divergence (Figure 2). As a secondary outcome, the sample was divided into a hyperdivergent group, a normodivergent group, and a hypodivergent group based on the value of the NSL/MP angle. Then, pairwise comparisons were made between the three groups: a parametric T-test for independent samples was used between groups that passed the normality test, while a non parametric Mann–Whitney U–test was used between the groups that unpassed the normality test.

Statistical significance was set at 0.05 for all the tests. 

## 3. Results

The Shapiro–Wilk test showed that the distributions of Δα were normal in all groups except for the hypodivergent group.

Table 2 showed the modification of OP in the whole sample and in the three groups:

- The whole sample showed overall a negligible modification of the OP (0.7°);
- Hyperdivergent patients presented an anterotation of the OP (3.422°);
- Hypodivergent patients presented a postrotation of the OP (−2.614°);
- Normodivergent patients showed a negligible modification of the OP (0.9333°).

Table 3 reports the pairwise comparisons between the three groups. A statistically significant difference was present between the hyperdivergent group and the hypodivergent group (6.036°; *p* < 0.05). No statistical differences were observed between the normodivergent and the hyperdivergent group (2.489°; *p* = n.s) and between the hypodivergent and the normodivergent group (3.547°; *p* = n.s.).

Table 4 shows how the increase of the skeletal divergence was related to a great modification of the OP.

The PFH/AFH% presented the same correlation with OP modifications (Table 4). 

## 4. Discussion

For the present investigation, only one aligner system was used to treat all patients in order to reduce confounding factors linked to mechanical properties of their material; it is demonstrated, indeed, that differences in thermoplastic material composition and thickness result in differences in flexibility, force activation and consequent stress-relaxation behavior [19]. According to some authors, the thermoforming process is the main factor in the decrease of thermal and mechanical properties, caused by a thickness reduction of aligners compared to the original dimension of thermoplastic foils, which results in lower forces expressed by the appliance [20]. However, the occlusal surface thickness reduction is rather consistent and reproducible [21,22].

To evaluate the orientation of maxillary occlusal plane on 3D models, the first step was to define a reference palatine plane, which was not a trivial aspect. In fact, to obtain repeatable measurements it is fundamental to identify stable anatomical structures with reduced interindividual variability and not influenced by orthodontic treatment. In the present study, it was decided to use the palatal rugae and the interincisal papilla as reference structures in the oral cavity. Batool Ali et al., in fact, concluded that the morphology of the palatal rugae is almost constant after various orthodontic treatments, including premolars extractions and REP, therefore could be considered a stable reference point [23]. Several authors have chosen inter incisive papilla as a stable palatine reference [24]. 

Regarding the bidimensional evaluation of the occlusal plane on lateral cephalograms, this represents the most commonly used method [24,25,26]. In the present study the OP on lateral cephalogram was identified as the bisected occlusal plane (BOP) as proposed by Downs: a line connecting the point that bisects the first molar cusp height and the point that bisects the incisal overbite [27]. 

The OP is of great importance for orthodontists: it is relevant during the diagnostic process, but also during treatment planning because it can be modified with orthodontic biomechanics, thus helping the resolution of the patient’s malocclusion. This is particularly evident in growing patients, where the presence of occlusal interference (i.e., bite blocks) may induce a new neuromuscular function, a modification of the maxillo-mandibular growth direction and a modification of the occlusal plane but also in adult patients, where OP modification can be usually observed following orthodontic treatment with fixed appliances [28].

Nevertheless, no information is available on the modification of OP in adult patients treated with CA. Some authors claim that a control over the OP on the vertical plane could be achieved with clear aligners through the bite block effect: the thickness of the aligners’ material combined with occlusal forces could lead to intrusion of the posterior teeth during treatment. Evidence of the bite block effect is provided by the occurrence of posterior open bites in patients after clear aligner therapy [11,12]. It is not clear, however, how these occlusal forces could act and if the introduction of clear aligners (i.e., bite blocks) in adults may alter the neuromuscular function, as some data from many researches seems to suggest [15,16,29]. Indeed, some clinical evidence for CAT shows an increase of clenching episodes frequency and an increase of muscles activity, probably with the aim of reducing the perception of nociceptive stimuli during orthodontic tooth movement. Some evidence reported that CAT induces a muscular soreness of limited clinical significance over 4 weeks [15,16]. Unfortunately, the available studies evaluated the modification of muscular function only during a short treatment period (from approximately 1 week to 3 months).

More data are available concerning the effects of occlusal splints on masticatory activity in adult patients over a longer time, which—considering the due differences and limitations—could help to understand what is observed with CAT. However, this topic is also not unambiguously clarified in the literature because of the different textures of splint compositions that are related to different muscles responses. Okeson found that soft splints produced a slight increase in the activity of muscles, especially masseter and temporalis, while on the contrary hard splints led to a decreased muscles activity [30]. Aligners used in CAT are made of a material that is more elastic and less rigid than conventional occlusal splints and not as soft as silicon splint and also thinner than conventional splints.

Some authors evaluated the response of masticatory muscles after removable thermoplastic orthodontic retainers during sleep. The retainers are commonly made of the same material used for aligners and for this reason the conclusions are currently more reliable than investigation regarding splints action. The effect of occlusal retention on masticatory muscle is still controversial. According to Manfredini et al., no significant effect of retainers on masticatory muscle activity during sleep was found, instead Castroflorio et al. reported that CAT was associated with an increase in masseter phasic contraction related to sleep bruxism. The limitations of those studies were related to not considering the different maxilla-mandibular vertical growth and the short observation period limited to only 3 months [31,32]. 

The authors of the present paper evaluated with 3D casts the modifications of OP in patients treated with CA over a period of 13 ± 5 months. Using stable palatal landmarks, the authors evaluated the effective modification of the OP at the end of CAT in three groups of vertical skeletal patients. In fact, it has been demonstrated that the inclination of the OP relates to the vertical facial pattern [33,34]. It is of common knowledge that three basic morphological types can be distinguished relative to the vertical plane: hyperdivergent, hypodivergent and normodivergent. Hyperdivergent are generally used to describe individuals with increased vertical dimension (long-face, dolichocephalic). Hypodivergent are characterized by reduced vertical dimension (short face, brachycephalic) and finally normodivergent have an average vertical dimension. They show, respectively, a vertical, a horizontal, and a neutral craniofacial growth [35]. Individuals with hyperdivergent patterns are characterized by a long anterior facial dimension and a short posterior facial dimension. Furthermore, they exhibit less bite force that is directly related to an excessive tooth eruption and a clockwise rotation of the mandible and the OP. Instead hypodivergents are defined by a short anterior facial dimension and a long posterior facial dimension. They also have a higher related bite force that results in molar intrusion and a counterclockwise rotation of the mandible and the OP [36].

Among the three groups studied (hypodivergent, hyperdivergent and normodivergent patients) the most important modification was observed in hyperdivergent patients (Δα = 3.42°). The mean difference between the hyperdivergent and the hypodivergent group was approximately 6.04° (*p* < 0,05). No statistically significant modifications were detected between hyperdivergent and normodivergent (2.49°, *p* > 0.05) and between hypodivergent and normodivergent patients (3.55°, *p* > 0.05). 

**Figure 3:**
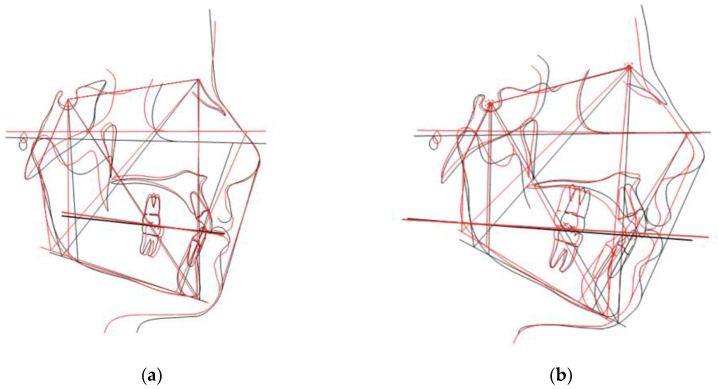
OP changes in (**a**) hypodivergent and (**b**) hyperdivergent patients in which pre-treatment is portrayed in black and post-treatment in red.

Table 2 shows an interesting finding: the OP showed a counterclockwise rotation in the hyperdivergent group and a clockwise rotation in the hypodivergent group (Figure 3).

In particular, in hyperdivergent patients, Δα increases after treatment because the distance between the palatine reference plane and the occlusal plane increases, especially at the level of premolars and molars. For this reason, the authors speak of anterotation of the occlusal plane with respect to the reference palatine plane. On the contrary, Δα decreases after treatment in hypodivergent patients. This indicates a reduction in the distance between the occlusal plane and the reference palatine plane and consequently a post-rotation of the occlusal plane with respect to the palatine reference plane. These data suggest that the CA had a different bite block effect in the two groups evaluated. 

It is known that the hyperdivergent patients have an OP that is often oriented downwards [36,37]. The CA produces a new muscular function that increases the vertical control and causes a more favorable orientation of the OP. Consequently, orthodontic aligners could be an effective alternative for the treatment of crowding in hyperdivergent patients because of the easier control of the vertical dimension. On the other hand, in hypodivergent patients the bite block effect of CAT, in association with greater muscle strength, could cause intrusion of upper molars and a post-rotation of the OP. The results of the present work suggest that CAT could not be the best choice for hypodivergent patients, where CAT alone could result in a worsening of the initial vertical dimension. 

The reason behind the observed effects on the OP could be the new occlusal contacts induced by CAT, translated into occlusal stimuli during swallowing, speaking, and mandibular movements. It is well known that hypodivergent patients present an occlusal barycenter anteriorly placed compared to hyperdivergent patients [36]. The different occlusal contacts and barycenter position stress the CA in a different way: in hypodivergent patients the aligners is most stressed in the anterior region while in hyperdivergent patients the aligners is most stressed in the posterior region. Clinicians may evaluate this aspect on aligners after 10 days of patient usage in most cases, observing a white area on the aligner surface (i.e., a mechanical stress zone) in the posterior region in hyperdivergent patients and in the anterior region in hypodivergent patients. It could be hypothesized that the occlusal stress modification induced by aligner may generate an immediate muscular modification (short term) and re-orientation of the OP (long term). 

The limitations of the present study are the small sample size and the fact that gender differences were not tested because the participants were predominantly female, limiting the generalizability of the study to all subjects. Moreover, the retrospective nature of sample recruitment could have led to a selection bias, although care was taken to avoid this limitation.

Further studies could include a larger number of patients to have a deeper insight into the effects of variables, such as gender and skeletal pattern. Moreover, it could be interesting to design a study that could analyze the causes that could explain the observed phenomenon.

Table 4 showed the correlation between the mean modification of OP, related to skeletal features. The authors showed how the vertical position of the mandible was related to an effective increase of the mean difference of Δα (rho 0.44, *p* < 0.05). In the same way the correlation with the Jaraback ratio showed how the Δα had an increase in mean difference in hyperdivergence and a reduction in mean difference in hypodivergent (rho −0.53, *p* < 0.01). 

## 5. Conclusions

The present study evaluated the occlusal plane changes after CAT using a novel 3D method, compared the results between groups with different craniofacial divergence as a secondary outcome. 

The results showed that CAT produced overall a slight and clinically not significant modification of the OP after a long treatment time of mean 13 months.

However, when dividing the sample into groups according to the type of facial divergence, the following results were observed:

- Normodivergent patients showed negligible changes of the OP;
- Hyperdivergent patients showed a counterclockwise rotation of the OP, which was favorable according to the general treatment plan;
- Hypodivergent patients showed a clockwise rotation of the OP;
- Absence of a control group; this is because it is a longitudinal study aiming to evaluate the modifications of the occlusal plane in a very limited time of approximately 13 months, so each subject is the control. It would have been interesting to have a control group composed of untreated patients but this would have raised ethical questions. Certainly, a control group could have been represented by patients in fixed therapy in which very heterogeneous biomechanics are used especially in hypo and hyperdivergent patients, however thus introducing some confounding factors. Moreover, it should also be considered that the effects of fixed therapy on the occlusal plane are already well described in the literature.

## Figures and Tables

**Table 1:** Cephalometric measurements.

Measurements	
Angular (°)	
NSL/MP	The angle between the sella nasion (SN) line and the mandibular plane (MP)
PP-MP	The angle between the palatine plane (PP), passing through Ans and Pns, and mandibular plane (MP)
PP-OP	The angle between the palatine plane (PP), passing through Ans and Pns, and occlusal Plane
OP-MP	The angle between the occlusal plane (OP), passing through the incisal edge of U1 to the midpoint of the U6 on the occlusal and mandibular Plane (MP)
PFH/AFH%	The ratio between the posterior facial height(S-Go) and anterior facial height (Na-Me) expressed in percentage

**Table 2:** The average Δα referred to the sample.

	Δα. Sample	Δα Hyperdivergent	Δα Hypodivergent	Δα Normodivergent
Mean	0.7	3.4	−2.6	0.9
Std. Deviation	5.5	6.6	3.9	4.7
Std. Error of Mean	1.1	2.3	1.5	1.5
Shapiro-Wilk	Yes	Yes	No	Yes

**Table 3:** Paired T-test and unpaired Mann–Whitney test used to evaluate correlation between Δα and vertical skeletal changes.

		*p* Value
Δα Hyperdivergent vs Δα Hypodivergent	Mann–Whitney	*p* < 0.05
Δα Hypodivergent vs Δα Normdivergent	Mann–Whitney	*p* > 0.05
Δα Hyperdivergent vs Δα Normodivergent	T Test	*p* > 0.05

**Table 4:** Pearson’s correlation test.

	Δα	NSL/MP	PP-MP	PP-OP	MP-OP	PFH/AFH%
Δα		0.4 * (0.03)	−0.02 (0.9)	−0.2 (0.4)	0.1 (0.5)	−0.5 ** (0.006)
NSL/MP	0.4 (0.03)		0.5 (0.01)	0.007 (0.9)	0.6 (0.33)	−0.8 (0.4)
PP-MP	−0.02 (0.9)	0.5 * (0.01)		0.5 (0.007)	0.7 (0.15)	−0.3 (0.2)
PP-OP	−0.2 (0.4)	0.007 (0.9)	0.5 (0.007)		−0.2 (0.3)	−0.05 (0.8)
MP-OP	0.1 (0.5)	0.6 (0.3)	0.7 (1.5)	−0.2 (0.3)		−0.4 (0.04)
PFH/AFH%	−0.5 ** (0.006)	−0.8 (0.4)	−0.3 (0.2)	−0.05 (0.8)	−0.4 (0.04)	

Rho (*p* value) * statistically significant with *p* < 0.05; ** statistically significant with *p* < 0.01.

## Data Availability

The data presented in this study are available on request from the corresponding author upon reasonable request.
